# Cardiac Papillary Fibroelastoma: Pulmonic Valve Involvement With Pulmonary Embolism and Pulmonary Hypertension

**DOI:** 10.7759/cureus.26302

**Published:** 2022-06-24

**Authors:** Danish Jilani, Mohammed Abuzahra, Meher B Ali

**Affiliations:** 1 Internal Medicine, Sinai Hospital of Baltimore, Baltimore, USA; 2 Cardiology, Sinai Hospital of Baltimore, Baltimore, USA; 3 Internal Medicine, Dow University of Health Sciences, Karachi, PAK

**Keywords:** pulmonary hypertension, pulmonary embolism (pe), primary cardiac tumor, heart valves, papillary fibroelastoma

## Abstract

Papillary fibroelastomas are rarely seen tumors of the cardiac valves. We present a case of papillary fibroelastoma present on the pulmonic valve. Diagnosis was made by characteristic echocardiogram findings. Complications of pulmonary embolism and pulmonary hypertension were present. Due to contraindications, the patient was managed with anticoagulation instead of surgery.

## Introduction

The incidence of primary cardiac tumors is estimated to be between 0.00017% and 0.033% [1]. They include myxomas, papillary fibroelastomas (PFE), rhabdomyomas, and fibromas. Papillary fibroelastomas are benign, pedunculated, and avascular tumors, derived from the endothelial cells. They account for three-fourths of all cardiac valve tumors [2]. Around 95% of these are present in the left-sided chambers of the heart [3]. They most commonly affect the aortic valve (35%), followed by the mitral valve (29%). Involvement of the tricuspid and pulmonic valves is rare (10% each) [4]. PFEs were previously diagnosed by only postmortem examinations; however, with better imaging modalities their premorbid detection is increasing. Here, we present a case of PFE which was diagnosed due to characteristic echocardiogram findings. Informed consent was taken.

## Case presentation

An 83-year-old female presented to us with shortness of breath due to heart failure exacerbation. She had a history of ischemic cardiomyopathy, atrial fibrillation, hypertension, pulmonary embolism, pulmonary hypertension, and obstructive sleep apnea. Her ejection fraction was 15%. She also had a biventricular implantable cardioverter-defibrillator placed due to sick sinus syndrome. Medications included aspirin, amiodarone, warfarin, furosemide, carvedilol and losartan.

**Figure 1:**
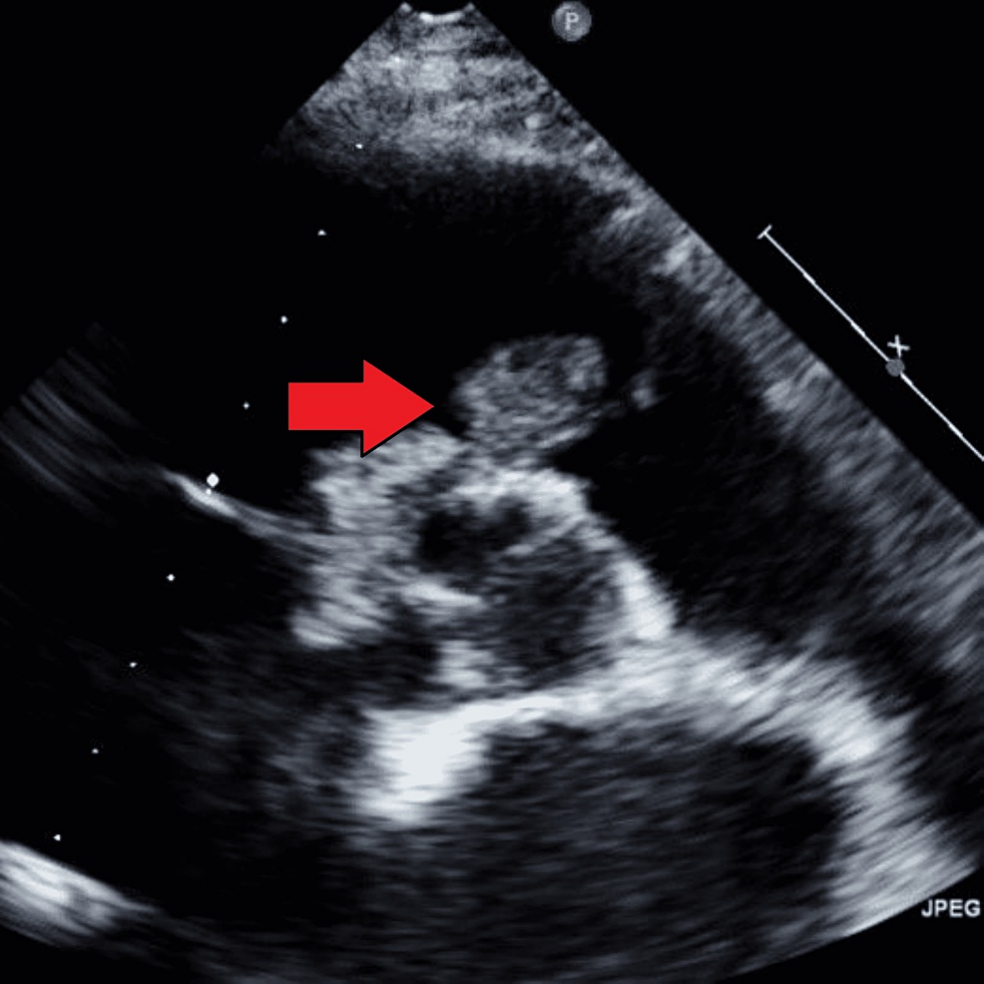
A pedunculated mass attached to the pulmonic valve is seen on a transthoracic echocardiogram.

**Figure 2:**
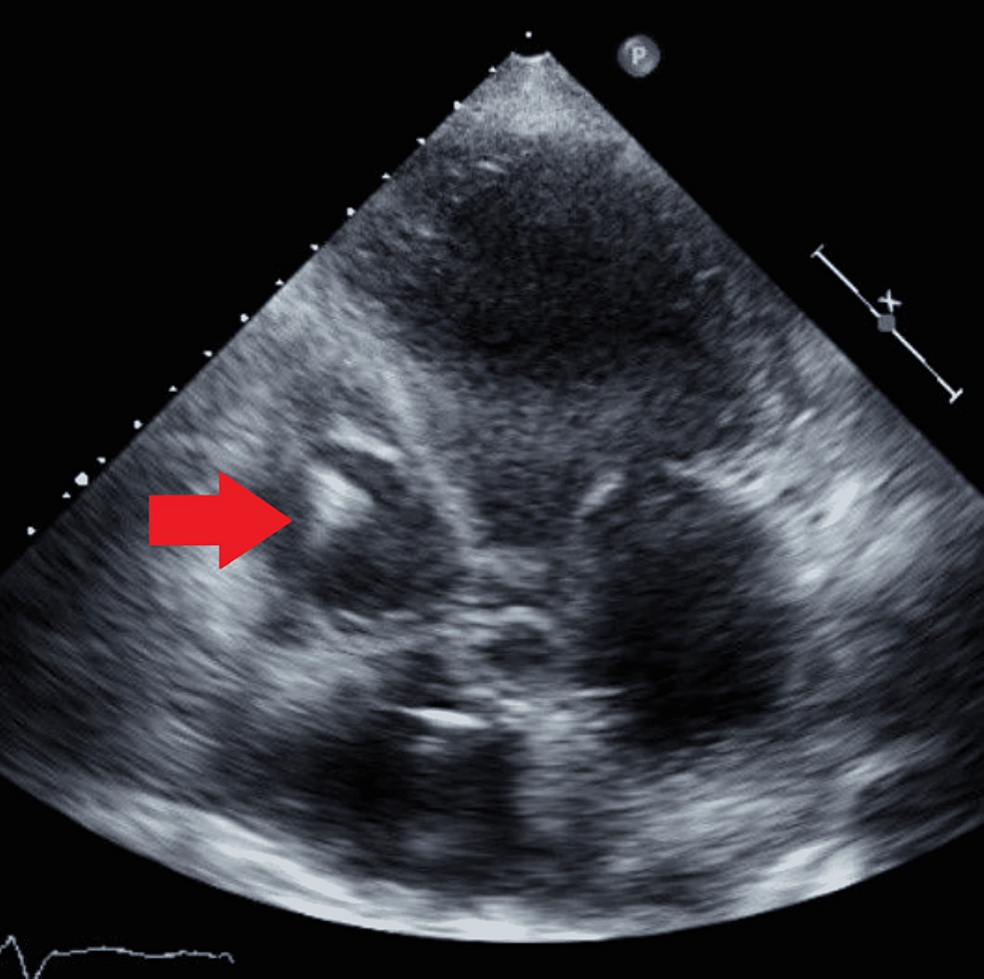
Papillary fibroelastoma can be seen in the right atrium.

**Figure 3:**
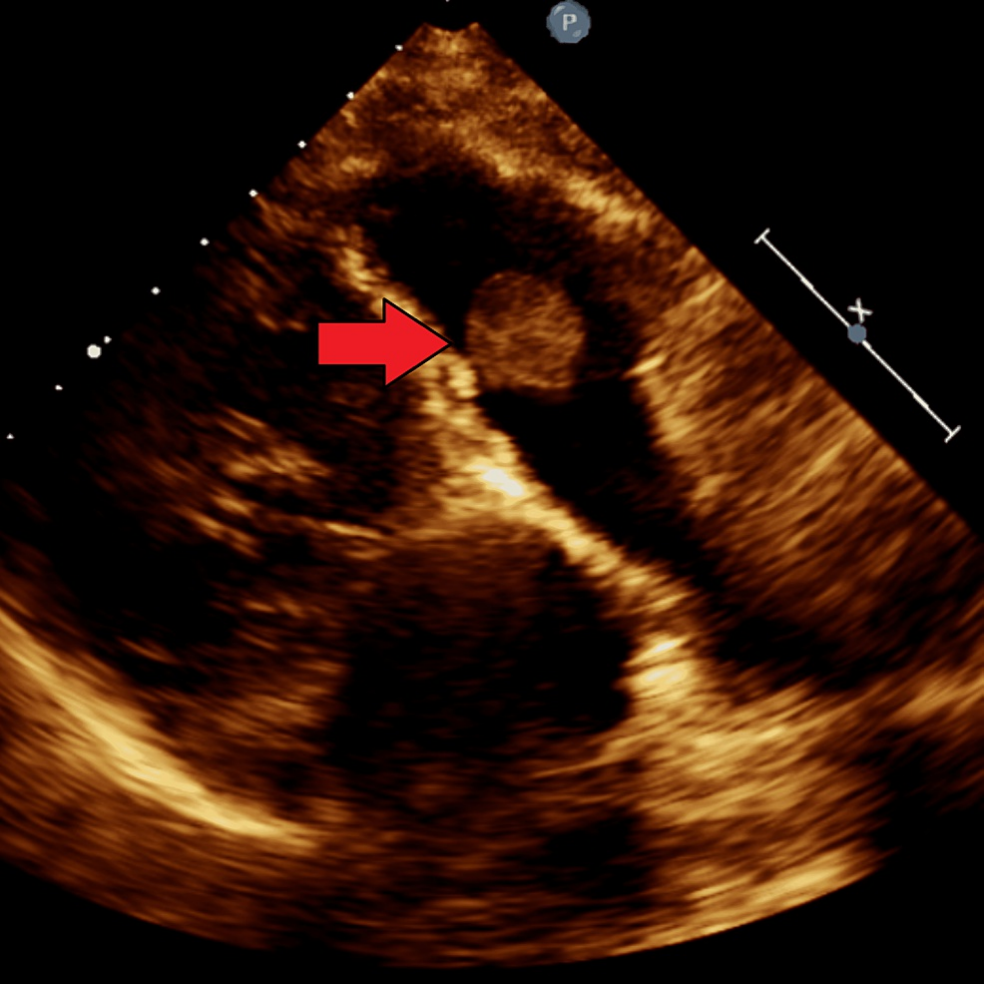
A well-circumscribed and homogenous mass can be seen on transthoracic echocardiogram.

On echocardiogram, a well-circumscribed and pedunculated mass was seen, which was attached to the ventricular aspect of the pulmonic valve leaflet. It measured 1.4x1.5 cm and prolapsed back-and-forth in the right ventricular outflow tract, without causing any obstruction (Figures 1-3). 

There was minimal pulmonic regurgitation and moderate pulmonary hypertension present. The mass was also visible on CT scan (Figure 2).

**Figure 4:**
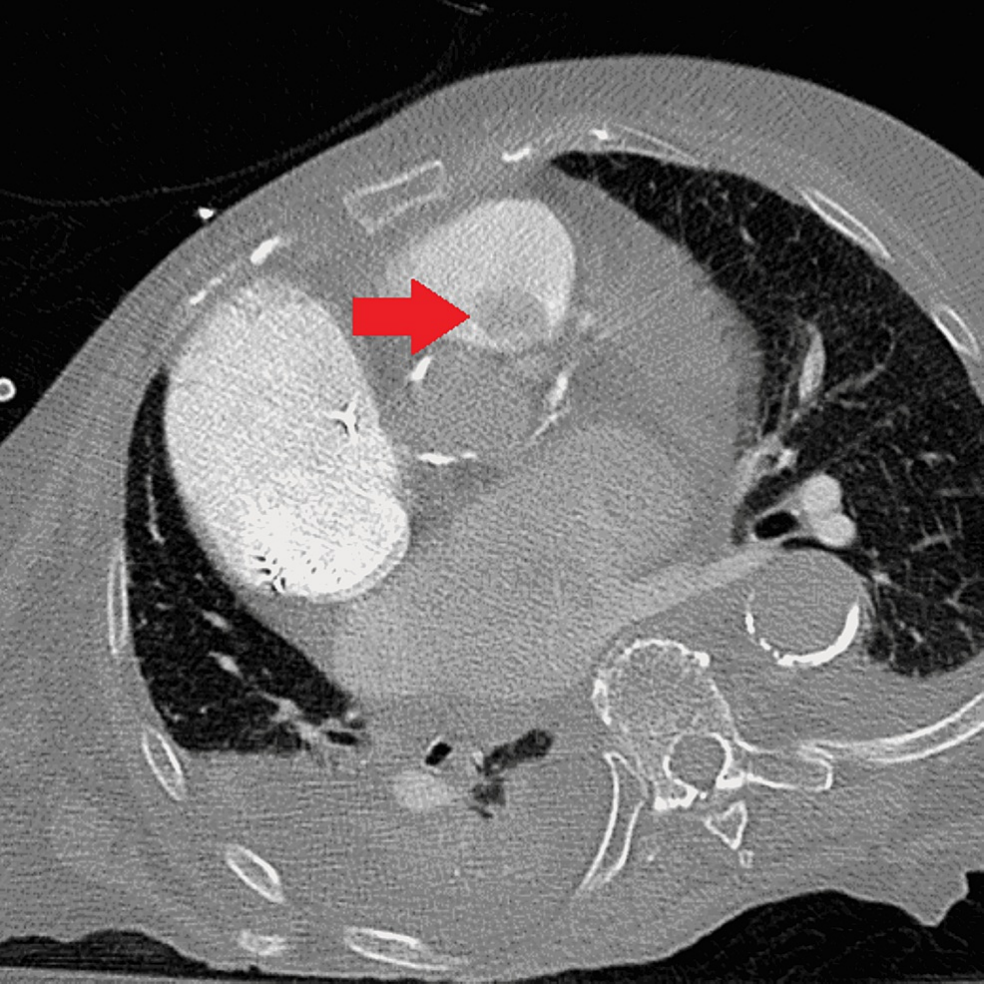
Computed tomography scan showing the papillary fibroelastoma.

Due to the characteristic imaging findings, a diagnosis of pulmonic valve PFE was made. The patient’s history of pulmonary embolism and pulmonary hypertension was presumed to be a complication of the PFE.

Given the patient’s multiple comorbidities, advanced age, and low ejection fraction, it was decided not to surgically intervene and she was maintained on anticoagulation. She was followed for five years due to multiple hospitalizations secondary to congestive heart failure exacerbation. During this time, the tumor was monitored on echocardiograms and it remained stable in size. She ultimately passed away after a complicated hospital course due to septic shock.

## Discussion

To diagnose PFEs, an echocardiogram is the initial imaging of choice. It has high sensitivity, specificity, and overall accuracy (88.9%, 87.8%, and 88.4%, respectively) [5]. Other diagnostic modalities include cardiac CT and MRI. MRI can be beneficial in determining vascularity [6]. Live 3D echocardiograms can also be used which helps provide additional information regarding the static and dynamic spatial location of the tumor. They are also used to discern neighboring structures [7]. A transesophageal echocardiogram should always be performed before surgical intervention for guiding the surgical approach [5].

On an echocardiogram, PFEs are small in size (usually 1.5cm) and mobile with a short pedicle or stalk attachment. They are well-circumscribed, homogenous, and sometimes have a speckled interior with stippling near the edges. They typically originate from the valvular surface of the heart [1,4,5]. Compared to PFEs, myxomas are primarily located in the atrium (75% left atrium and 15-10% right atrium), are larger, have a heterogeneous appearance, and are attached by a longer stalk to the interatrial septum [8]. Grossly upon resection, the tumor has a sea anemone-like appearance with multiple papillary fronds. On histopathology, it is covered by endothelium, with a mucopolysaccharide and vascular core. This core contains variable amounts of collagen, smooth muscle, and elastic fiber [9].

If left untreated, PFEs can embolize to cause a stroke if left-sided, and pulmonary embolism and pulmonary hypertension if right-sided. Coronary ostial occlusion may be seen in larger-sized or highly mobile masses. Acute valvular dysfunction may be present; however, valvular insufficiencies are not commonly seen [5]. Definite management is not agreed upon. Surgical excision of the tumor is recommended for all patients with prior embolic complications and may also be offered to asymptomatic patients due to the risk of embolization. Recurrence after surgery has not been reported. If a patient is not a surgical candidate, anticoagulation can be considered to prevent embolism.

## Conclusions

PFEs are rare tumors of the heart, most commonly seen on the aortic valve. Our patient had a pulmonic valve PFE, which was diagnosed due to the characteristic imaging findings present on the echocardiogram. These tumors are small, mobile, well-circumscribed, and homogenous with a speckled interior. In this case, complications included pulmonary embolism and pulmonary hypertension. Though surgery is recommended in patients with PFE, our patient had multiple comorbidities and was maintained on anticoagulation.
